# Accuracy of Transvaginal Ultrasound in the Diagnosis of Intrauterine Lesions

**DOI:** 10.1055/s-0041-1732462

**Published:** 2021-08-30

**Authors:** Adriana Elisa de Miranda Murta Pereira, Junia Franco, Fernanda Silveira Machado, Selmo Geber

**Affiliations:** 1 Department of Obstetrics and Gynecology, Universidade Federal de Minas Gerais, Belo Horizonte, MG, Brazil

**Keywords:** transvaginal ultrasound, hysteroscopy, intrauterine lesions, accuracy, histeroscopia, lesões intrauterinas, ultrassom-transvaginal, acurácia

## Abstract

**Objective** To evaluate the accuracy of transvaginal ultrasound in the diagnosis of intrauterine lesions, using hysteroscopy as the gold standard.

**Methods** This was a prospective observational study with 307 patients. All patients underwent hysteroscopy after a previous transvaginal ultrasound to compare the results. The hysteroscopy was performed by experienced examiners, and transvaginal ultrasounds were performed in various public and private services, which is reflective of routine healthcare practices in obstetrics and gynecology. The sensitivity, specificity, and accuracy of the transvaginal ultrasound were calculated using hysteroscopy as the gold standard. The level of agreement between the two exams was calculated using the Kappa test.

**Results** The mean age was 56.55 ± 12.3 years. For endometrial polyps, we observed a sensitivity of 39.8%, specificity of 72.7%, accuracy of 52.8%, and Kappa index of 0.11 ( *p* = 0.025). For fibroids, the sensitivity was 46.7%, specificity was 95.0%, accuracy was 87.9%, and Kappa index was 0.46 ( *p* < 0.001). For endometrial thickening, the sensitivity was 68.7%, specificity was 41.7%, accuracy was 47.6%, and Kappa index was 0.06 ( *p* = 0.126). For endometrial atrophy, we found a sensitivity of 6.7%, specificity of 99.3%, accuracy of 90.2%, and Kappa index of 0.10 ( *p* = 0.006). For the other findings, the sensitivity was 15.6%, specificity was 99.6%, accuracy was 87.3%, and Kappa index was 0.23 ( *P* < 0.001).

**Conclusion** Our study demonstrated a low level of accuracy of transvaginal ultrasound for the diagnosis of endometrial lesions, when performed by a non-experienced professional. Thus, it is important to consider the use of hysteroscopy to avoid unnecessary and inappropriate treatments.

## Resumo

**Objetivo** Avaliar a acurácia do ultrassom transvaginal para o diagnóstico de lesões intrauterinas, tendo a histeroscopia como padrão de referência.

**Métodos** Foi realizado um estudo observacional prospectivo em 307 pacientes, submetidas à histeroscopia após ultrassonografia prévia para comparação dos resultados. A histeroscopia foi realizada por duas médicas com experiência, e os exames de ultrassom foram realizados em diversas fontes, públicas ou privadas, como ocorre no cotidiano da assistência à saúde em nosso meio. Foram avaliados sensibilidade, especificidade e acurácia, tendo a histeroscopia como padrão-ouro. O nível de concordância foi avaliado pelo teste de Kappa.

**Resultados** A idade média foi de 56,55 ± 12,3 anos. Os resultados para pólipo endometrial foram: sensibilidade 39.8%, especificidade 72,7%, acurácia de 52,8%, e índice Kappa 0,11 ( *p* = 0,025). Para mioma, sensibilidade 46,7%, especificidade 95,0%, acurácia 87,9%, e índice Kappa 0,46 ( *p* < 0,001). Para espessamento endometrial, sensibilidade 68,7%, especificidade 41,7%, acurácia 47,6%, e índice Kappa de 0,06 ( *p* = 0,126). Para atrofia, sensibilidade 6,7%, especificidade 99,3%, acurácia 90,2%, e índice Kappa 0,10 ( *p* = 0,006). Para outros achados, sensibilidade 15,6%, especificidade 99,6%, acurácia 87,3%, e índice Kappa 0,23 ( *p* < 0,001).

**Conclusão** Nosso estudo demonstrou baixo nível de acurácia da ultrassonografia transvaginal para o diagnóstico de lesões endometriais, quando realizada por profissional não experiente. Assim, é importante considerar o uso da histeroscopia para evitar tratamentos desnecessários e inadequados.

## Introduction

Among all examination methods available to evaluate the uterine cavity, two-dimensional (2D) transvaginal ultrasound (TVUS) is the most widely used. 1 It is easy to perform, noninvasive, inexpensive, and it is the most accessible method available for the general population. 1 2 3 However, some studies have demonstrated that ultrasound should not be the only method used for intrauterine evaluation, as it often does not accurately differentiate between intrauterine lesions. 4 5 6 7 8 9

Capmas et al. 6 evaluated postmenopausal women with endometrial thickening and found a weak correlation between ultrasound diagnosis and hysteroscopy results, concluding that ultrasound could not be used as the sole diagnostic method when evaluating the uterine cavity. Goyal et al., 10 assessed women with abnormal uterine bleeding and concluded that when no endometrial alterations are identified on ultrasound, there is no need to complement this approach with other methods. However, in the presence of intrauterine alterations, another evaluation is necessary due the low specificity. These studies have shown that ultrasound is useful as an initial method of analysis when examining uterine cavity pathologies, but it cannot be the only method used. Loverro et al. 11 evaluated the accuracy of ultrasound diagnosis when compared with hysteroscopy and endometrial biopsy among women with postmenopausal uterine bleeding. They observed that ultrasound was not very accurate when evaluating intrauterine lesions. Yela et al. 5 also described that ultrasound was not very accurate when compared with hysteroscopy in the evaluation of endometrial diseases.

Conversely, other studies have shown that good diagnostic accuracy was achieved with TVUS when identifying intrauterine lesions. 2 8 However, although they described high sensitivity and specificity for some lesions, such as polyps, fibroids, and septum, they also described low sensitivity and specificity for adhesions, endometrial thickening, and unicornuate uterus.

Considering the above, we understand that there is currently no consensus regarding the accuracy of ultrasound when evaluating intrauterine lesions. Given the prominence of ultrasound as a diagnostic method, it is important to evaluate the actual accuracy and reliability of this method, when used in routine diagnostics. Furthermore, there is a need to detect the possible reasons underlying the differences observed between various studies. Hysteroscopy is the method of choice for intracavitary evaluation, as it allows a direct view and evaluation of the uterine cavity. 4 5 7 12 It is, thus, the gold standard when assessing the accuracy of ultrasound examinations. 4

The aim of our study was to evaluate the accuracy of TVUS performed at non-specialized centers when diagnosing intrauterine lesions in women at all stages of reproductive life, based on the hysteroscopic diagnosis.

## Methods

We performed a cross sectional, prospective study from April 2017 to September 2018 at the Hospital das Clínicas of Universidade Federal de Minas Gerais. The study was approved by the local research ethics committee (COEP-UFMG – CAAE 68632517.6.0000.5149–Acceptance Protocol Number 2237639–08/24/2017). All women read and signed a written informed consent form. We evaluated 364 patients who had undergone a previous endovaginal ultrasound examination and were diagnosed with intrauterine disease. All patients were referred for hysteroscopic evaluation to confirm, or not, the ultrasound findings. The following intracavitary changes were considered: endometrial polyps, endometrial thickening (≥ 5 mm at postmenopause), submucosal fibroids, endometrial atrophy, and other findings, including septal or synechia changes, and a uni or bicornuate uterus. At the end of the study, we compared the results obtained via hysteroscopy with those obtained by TVUS.

## Ultrasound

All patients included in the study underwent 2D TVUS. The examination reports were evaluated irrespective of the examiner or equipment used. Thus, we did not evaluate an individual's capability to conduct an assessment, but rather explored the overall method.

## Hysteroscopy

All hysteroscopic examinations were performed on an outpatient basis, following the same method and routine. All patients received oral analgesic medication 1 hour before the procedure, and no local anesthetic or sedation was used. A rigid hysteroscope with a 30° rigid-angle optics (model number 20212120) (Karl Storz, Tuttlingen, Germany) and Bettocchi-type sheaths were used. As a distension medium, we used 0.9% saline solution coupled to the hysteroscopy set's irrigation system.

## Statistical Analysis

Sensitivity, specificity, false-positive, and false-negative values were used to assess the accuracy of the TVUS examination for each of the variables studied. Hysteroscopy was used as the gold standard. Agreement between the two types of exams was also evaluated, based on the results of the presence or absence of a given lesion, and reported as the Kappa index. The exams were considered concordant when *p* < 0.05 and the degree of agreement ranged from 0 to 1; the level of agreement was stronger when it was closer to 1.

## Results

A total of 364 patients were evaluated, and 57 were excluded. Thirty-eight had severe cervical stenosis and 2 had severe obesity, which precluded office hysteroscopy; 2 had cervical cancer, 15 had other diagnosis by hysteroscopy that were not evaluated in the present study. Therefore, 307 patients were included in the study. The mean age was 56.55 ± 12.3 years. There were no complications, such as uterine perforations, heavy bleeding, severe vasovagal reactions, or disabling pain. Patients with soft cervical stenosis at the time of the examination (108) had mild bleeding and experienced mild pain, which did not preclude them from undergoing the examination. No patients had to be hospitalized after the procedure. A total of 15 patients (4.8%) had a mild vasovagal reaction, without impediment to the exam. The exam execution time was ∼ 15 minutes for each patient, from the moment they laid down to the moment they got up. From the 307 patients included, we got a total of 339 ultrasound diagnoses and 373 hysteroscopy results ( Table 1 ), representing a possibility of more than one lesions for each patient.

**Table 1:** Comparison of total diagnoses obtained by transvaginal ultrasound and hysteroscopy for each type of lesion evaluated

DIAGNOSES	TVUS	HYSTEROSCOPY
Polyp	107	186
Fibroid	34	45
Endometrial thickening	186	67
Endometrial atrophy	4	30
Others	8	45
Total	339	373

We identified 150 corresponding diagnoses between TVUS and hysteroscopy within the 339 ultrasound diagnoses. This indicates that 44.24% of the findings yielded concordant diagnoses. The sensitivity, specificity, false-positive and false negative values, and overall accuracy of the ultrasound examination for each of the intrauterine lesions, considering hysteroscopy as the gold-standard, can be observed in Table 2 . When we performed the agreement analysis between the two examination methods, we observed agreement across all evaluated lesions, except for endometrial thickening. However, the level of agreement was not strong ( Table 3 ).

**Table 2:** Sensitivity, specificity, and accuracy of transvaginal ultrasound in the evaluation of intrauterine lesions based on the diagnosis obtained by hysteroscopy

Lesion	Sensibility	Specificity		False positive	False negative	Accuracy
**Polyp**	39.8%	72.7%		30.8%	56%	52.8%
**Fibroid**	46.7%	95%		38.2%	8.8%	87.9%
**Endometrial thickening**	68.7%	41.7%		75.3%	17.4%	47.6%
**Endometrial atrophy**	6.7%	99.3%		50%	9.2%	90.2%
**Others**	15.6%	99.6%		12.5%	12.7%	87.3%

**Table 3:** Analysis of agreement of results obtained by transvaginal ultrasound and hysteroscopy in the evaluation of intrauterine lesions

Lesion	Kappa Cohen's analysis	*p* -value
Polyp	0.11	0.025
Fibroid	0.46	< 0.001
Endometrial thickening	0.06	0.126
Endometrial atrophy	0.10	0.006
Others	0.23	< 0.001

## Discussion

Our study identified weak and moderate levels of agreement between ultrasound and hysteroscopy diagnoses for endometrial lesions. In cases of endometrial thickening, there was no agreement. As the study group consisted of women ranging in age from 23 to 89 years, we could evaluate women of reproductive age and postmenopausal women.

Hysteroscopy was used as the gold standard, as it allowed for the direct observation of the uterine cavity and is associated with a high level of accuracy, as described previously. 4 5 9 12 13 14

When evaluating the diagnosis of fibroids, we identified an accuracy level of 87.9%, but this was associated with very low sensitivity, specificity, and a low Kappa index. The Kappa index value was similar to that observed by Vercellini et al., 15 who examined 793 women with menorrhagia. Regarding the accuracy of the method used, our results were similar to those described by Wanderley et al., 9 who studied 191 women who also underwent ultrasound examinations at various healthcare centers. However, our results were inferior to those reported by Veena and Shivalingaiah, 8 who described an accuracy rate of 96.7%. Regarding sensitivity and specificity, Niknejadi et al. 2 evaluated the results of 643 infertile women, and they observed greater sensitivity than was described in our study (89.2%); they also found a similar specificity (92.5%). These differences can be explained by the limited number of patients analyzed (8) and the fact that the examinations were performed by the same examiner. 2

With respect to the diagnosis of polyps, we identified a low accuracy rate, low sensitivity and specificity, and a low Kappa index. Our results were lower than those previously described by other authors, who performed their examinations at the same center and with the same equipment. 2 3 4 8 However, our results were similar to those described by Wanderley et al., 9 who conducted the study irrespectively of the examiner or equipment.

When evaluating anatomical changes of the uterine cavity, we identified a high level of accuracy and specificity, and a low sensitivity and Kappa index. To the best of our knowledge, only one study studied anatomical changes of the uterus, yet the results were presented separately for each lesion. 2 The authors described similar specificity levels; however, the sensitivity observed in our study was lower. This difference can be explained by the lower number of cases evaluated in our study, and by the fact that the ultrasound exams were performed by the same examiner in the previous study.

When we evaluated the diagnosis of endometrial atrophy, we identified a high degree of accuracy and specificity, as well as a low sensitivity and Kappa index. Our results are similar to those described by Babacan et al. 3 When evaluating endometrial thickening, we observed an accuracy of 47.6%, a sensitivity of 68.7%, and a specificity of 41.7%. Our results were lower than those observed by Veena and Shivalingaiah, 8 who reported an accuracy of 88.33%; Wanderley et al., 9 who described an accuracy of 63.2%; and Niknejadi et al., 2 who described a sensitivity of 56.2% and a specificity of 99.6%. Even though we found low accuracy for other lesions, this was the only diagnosis for which there was no agreement between the two exams approaches. As many cases of endometrial polyps diagnosed through hysteroscopy were initially identified as suspected endometrial thickening on ultrasound, we understand that this may be an important limitation of ultrasound examinations.

To ensure that the study findings are applicable to everyday clinical practice, we included all ultrasound examinations performed at non-specialized centers, regardless of examiner or equipment. This fact created a bias as we did not control intra-examiner and intra-equipment variations; however, it rendered more reliable results when analyzing routine clinical examinations. Certainly, examinations performed at centers with an experienced examiner and state-of-the-art equipment would yield different results from those observed here.

## Conclusion

Our study demonstrated a low level of accuracy for TVUS for the diagnosis of endometrial lesions, when performed by a non-experienced professional. Thus, it is important to consider the use of hysteroscopy to avoid unnecessary and inappropriate treatments. Moreover, it is important to continue training professionals to improve diagnostic accuracy.
